# Quality of life in rehabilitation outpatients: normal values and a comparison with the general Dutch population and psychiatric patients

**DOI:** 10.1007/s11136-015-1060-1

**Published:** 2015-07-10

**Authors:** Ernst Schrier, Irene Schrier, Jan H. B. Geertzen, Pieter U. Dijkstra

**Affiliations:** Department of Rehabilitation Medicine HPC:CB40, Centre for Rehabilitation, University Medical Centre Groningen, University of Groningen, Hanzeplein 1, 9713 GZ Groningen, The Netherlands; Sinai Centre, Amersfoort, The Netherlands; Department of Oral and Maxillofacial Surgery, University Medical Centre Groningen, University of Groningen, Groningen, The Netherlands

**Keywords:** Quality of life, Rehabilitation centre, Outcome assessment, Rehabilitation outpatients

## Abstract

**Purpose:**

To provide Dutch normal values for rehabilitation outpatients with chronic pain or musculoskeletal diseases utilizing the World Health Organization Quality of Life questionnaire abbreviated version (WHOQOL-BREF) and analyse influence of diagnosis and patient characteristics on normal values and increase understanding in those values.

**Methods:**

Five hundred and forty-two outpatients were referred to a rehabilitation psychologist. Referral diagnoses were “musculoskeletal”, “chronic pain”, “neurological” and “miscellaneous”. Comparisons between groups were made for each of the four domains of the WHOQOL-BREF (scoring range 4–20).

**Results:**

Domain scores of rehabilitation outpatients were physical domain 11.0 (±2.7), psychological domain 13.6 (±2.4), social domain 14.8 (±3.4) and environmental domain 14.2 (±2.2). Outpatients with chronic pain reported the lowest scores on the WHOQOL-BREF when compared to the “musculoskeletal”, “neurological” and “miscellaneous” groups. Increased age, lower education, living alone and unemployment had a negative impact on WHOQOL-BREF scores. Compared to the general Dutch population, rehabilitation outpatients scored, unadjusted for age, significantly lower difference for the physical domain 4.5 [95 % confidence interval (CI) 4.2; 4.8], the environment domain 1.7 (95 % CI 1.5; 2.0), the psychological domain 1.1 (95 % CI 0.4; 1.2) and the social domain 0.4 (95 % CI 0.0; 0.8).

**Conclusions:**

WHOQOL-BREF scores of rehabilitation outpatients are lower and differed significantly from normal values of a Dutch population in all four domains. Therefore, the WHOQOL-BREF can be used to measure the subjective impact of their disease or injury. The subjective impact of chronic pain was found to be particularly high.

## Introduction

Due to modern health care, more and more patients with potentially lethal diseases are cured or disease progression is reduced [1]. Therefore, the treatment goals of patients in rehabilitation have shifted from how to survive into how to adapt to and cope with a chronic disease [2]. In the last decades, the patient’s perspective on the pros and cons of treatment has grown in importance, resulting in increased attention for the impact of (chronic) disease or injury on patient’s quality of life (QOL). QOL can be assessed utilizing the WHOQOL-BREF [3], in which QOL is defined as “an individual’s perception of their position in life in the context of the culture and value systems in which they live and in relation to their goals, expectations, standards and concerns”. Domain scores are scaled in a positive direction (i.e. higher scores denote higher QOL).

It should be noted that apart from disease and injury, QOL is also influenced by social functioning [4, 5], education, employment [6], comorbidity [7], self-efficacy [8] and goal adjustment [9]. Furthermore, both gender and age influence QOL; women score significantly higher on the social domain of QOL and lower on all the other domains of QOL compared to men [10]. Finally, QOL has been shown to decrease with increasing age [11]. A decreased QOL is found in patients with a somatic disease as well as in patients with a psychiatric disorder [4, 12–14]. In the latter case, QOL is inversely related to severity of psychopathology [4, 7].

The negative influence on the QOL by somatic and psychiatric diseases is found in all domains. This influence is well understood since Engel introduced the biopsychosocial model [15]. This model is the foundation of the multidisciplinary treatment approach in rehabilitation. Today the International Classification of Functioning (ICF) is adopted as a framework for rehabilitation, and an important goal in rehabilitation is to increase QOL of patients [16, 17]. Currently no normal values for QOL of Dutch rehabilitation outpatients are available, which are essential for a correct comparison between rehabilitation outpatients, the general Dutch population and psychiatric outpatients. Normal values for rehabilitation outpatients provide insight into whether the instrument can measure the impact and variations of a disease or injury on the QOL.

The aims of this study were to provide normal values for Dutch rehabilitation outpatients with chronic pain or musculoskeletal diseases utilizing the WHOQOL-BREF, to analyse the influence of diagnosis and patient characteristics of rehabilitation outpatients on normal values and to compare normal values with those of the general Dutch population and psychiatric outpatients.

## Method

### Patients

Between January 2008 and January 2013, 607 outpatients from the Department of Rehabilitation Medicine of the University Medical Centre Groningen (UMCG) were referred to a psychologist. They were referred by a rehabilitation specialist for a psychological assessment and/or treatment. Prior to this assessment, a set of questionnaires and a consent form were sent by mail to the patients with a request to fill out all forms. During the assessment, a semi-structured interview was conducted to determine a treatment plan. During the intake procedure, patient’s gender, age, educational level, employment and marital status were collected. The rehabilitation specialist’s referral medical diagnosis was retrieved from the medical records.

### Reference groups

The general Dutch population reference group was based on the Dutch manual WHOQOL-100 and WHOQOL-BREF. This group of 626 persons had a mean age of 53.9 (SD 16.2), and 67.5 % of the group were women [18].

The psychiatric reference group consisted of 410 psychiatric outpatients with a mean age of 33.5 (SD 8.3), and 58.8 % of the group were women. It was a mixed diagnostic group: 54 persons who did not obtain a DSM-IV diagnosis, 224 with a single axis diagnosis and 132 with a diagnosis on axis 1 as well as axis 2 [7].

### Instruments

The WHOQOL-BREF is a condensed version of the WHOQOL questionnaire. The WHOQOL-BREF is a 26-item questionnaire that correlates well with the original 100-item questionnaire (*r* ranges between 0.88 and 0.96) [19]. It assesses the individual’s perceptions in the context of his/her culture and value system, personal goals, standards and concerns. The WHOQOL instruments were developed collaboratively in a number of centres worldwide and have been field tested widely [20]. Of the 26 items, 24 items were used to calculate the four QOL domains: physical health (7 items), psychological (6 items), social relationships (3 items) and environment (8 items). Transformed domain scores range from 4 to 20. A higher score indicates a better QOL. The two remaining items, sometimes used to calculate overall QOL and health, were not used in this study as recommended by the WHO.

### Analysis

Data were anonymized and analysed using IBM SPSS Statistics (v.20). P–P and Q–Q plots were used to assess the normal distribution of the dependent variables. Results are significant at *p* ≤ 0.05 unless stated otherwise. A Pearson Chi-square test and ANOVA were used to determine whether gender, marital status, education, employment and age differed between the referral diagnosis groups. The dependent variables in the current study were the scores on the four domains of the WHOQOL-BREF. The WHOQOL-BREF scores of the referral diagnosis groups were compared using a one-way ANOVA. A series of Tukey’s post hoc tests were used for pair-wise comparisons. For regression analyses, several dummy variables were computed. Education was dichotomized into low education (1 = low and lowest, 0 = middle and high) according to the International Standard Classification of Education (ISCED) 2011. Low education equals the ISCED level 0–4, middle the level 5 and high the level 6–9 [21]. Social status was dichotomized into living alone (0 = living alone, 1 = living with the family or a partner), referral diagnosis was dichotomized into chronic pain (1 = chronic pain, 0 = musculoskeletal, neurological and miscellaneous), and employment was dichotomized as follows (0 = retired, unemployed, student, welfare, 1 = work, sick leave compensation). In the Dutch society, persons who are on sick leave keep their job for at least 2 years and get between 70 and 100 % financial compensation, and for this reason, sick leave compensation was counted as work. To analyse the influence of gender, age, education, social status, employment and diagnosis, a hierarchical stepwise regression analysis was applied for each domain of WHOQOL-BREF. To compare differences in means of rehabilitation outpatients with a general Dutch population and psychiatric outpatients [4], confidence intervals (CI) for differences in means were calculated for each domain, unadjusted for age and or gender, since data on a personal level of the reference groups were not available [22].

## Results

In total, 65 patients were excluded from the current study (11 %), 32 did not sign informed consent, 18 were under 18 years of age, and 15 were excluded because of missing data resulting in 542 potential participants in the current study.

Four referral diagnosis groups were specified, based on the diagnosis treatment combination used in the Netherlands to categorize patients for funding purposes, and this method is used in all Dutch rehabilitation centres.

The first referral diagnosis group was “musculoskeletal” including “disease or injury of the upper extremity” and “other musculoskeletal diseases” (*n* = 280, 52 %). The second referral diagnosis group was “chronic pain” including patients with chronic pain (*n* = 174, 32 %). The third referral diagnosis group was “neurological” including “diseases or injury of the central nerve system” or “peripheral nerves” (*n* = 59, 11 %), and the last group is a miscellaneous group (*n* = 29, 5 %) (Table 1). A benchmark was made in 2012 of all treatments (*n* = 103410) in 20 Dutch rehabilitation centres, according to the same categories. Brain injury patients were the largest group (32 %) followed by musculoskeletal (24 %), chronic pain (17 %), neurology (13 %), organs (6 %), paraplegic (5 %) and amputations (3 %) in that benchmark [23]. In our study in outpatients, brain injury was rare, but the other three most important diagnosis groups had a similar distribution. Because the same method to diagnose was used, we expect that our sample is representative of at least musculoskeletal group and chronic pain group.

**Table 1:** Referral diagnosis of the rehabilitation specialist and grouping of patients in the current study

Diagnosis	Division of the groups	*n*
Musculoskeletal diseases	Musculoskeletal	280
Chronic pain	Chronic pain	174
Neurology	Neurological	59
Brain injury	Miscellaneous	7
Paraplegic	Miscellaneous	2
Amputations	Miscellaneous	16
Organs	Miscellaneous	4
Total		542

In total, 68 % of the patients were female; 88 % had an age between 20 and 60 years. A majority of patients were living with a partner (67 %), 11 % lived with their parents, 22 % lived alone, and 56 % were employed (Table 2).

**Table 2:** Characteristics of participants according to referral diagnosis of the rehabilitation specialist

	Total group (*n* = 542)	Musculoskeletal (*n* = 280)	Chronic pain (*n* = 174)	Neurological (*n* = 59)	Miscellaneous (*n* = 29)	*p* value
	*n* (%)	*n* (%)	*n* (%)	*n* (%)	*n* (%)	
Female	366 (67.5 %)	196 (70.0 %)	116 (66.6 %)	39 (66.1 %)	15 (51.7 %)	0.241^a^
Education						0.313^a^
Low/lowest	198 (36.5 %)	97 (34.6 %)	73 (42.0 %)	20 (33.9 %)	8 (27.6 %)	
Medium	211 (38.9 %)	113 (40.4 %)	63 (36.2 %)	23 (39 %)	12 (41.4 %)	
High	133 (24.6 %)	70 (25.0 %)	38 (21.8 %)	16 (27.1 %)	9 (31 %)	
Social status						0.657^a^
Alone	121 (22.3 %)	57 (20.4 %)	41 (23.6 %)	12 (20.3 %)	11 (37.9 %)	
With parents	58 (10.7 %)	31 (11.0 %)	17 (9.8 %)	9 (15.3 %)	1 (3.4 %)	
With partner	363 (67.0 %)	192 (68.6 %)	116 (66.6 %)	38 (64.4 %)	17 (58.6 %)	
Employed	302 (55.7 %)	168 (60.0 %)	96 (55.2 %)	25 (42.4 %)	13 (44.8 %)	0.051^a^
Age, mean (SD)	41.0 (14.0)	40.3 (14.2)	41.7 (14.0)	41.8 (12.8)	43.7 (15.6)	0.491^b^

^a^Chi-square test

^b^ANOVA

Gender [*χ*^2^ (*df* 3, *n* = 542) = 4197, *p* = 0.241], marital status [*χ*^2^ (*df* 6, *n* = 542) = 7.088, *p* = 0.313], education [*χ*^2^ (*df* 6, *n* = 542) = 4144, *p* = 0.657] and employment [*χ*^2^ (*df* 3, *n* = 542) = 7,755, *p* = 0.051] did not differ significantly between the different diagnosis groups. Employment was almost a significant difference between groups, and most deviant were the neurological group and the miscellaneous group. The four domains of the QOL were normally distributed. Cronbach’s alpha for the WHOQOL-BREF was 0.90. Removing items from the questionnaire resulted in lower values of alpha.

Compared to the total group rehabilitation outpatients, the chronic pain group scored significantly lower in every domain except the environment, and the musculoskeletal group scored significantly higher in all four domains. There is a significant difference between the musculoskeletal group and the chronic pain group in all four domains (Table 3).

**Table 3:** Comparison of WHOQOL-BREF domains between the four diagnosis groups of rehabilitation outpatients included in the University Medical Centre Groningen between 2008 and 2012

Domain	Total group outpatients, *n* = 542 Mean (SD)	Musculoskeletal, *n* = 280 Mean (SD)	Chronic pain, *n* = 174 Mean (SD)	Neurological, *n* = 59 Mean (SD)	Miscellaneous *n* = 29 Mean (SD)	One-way between-groups ANOVA *p* value
Physical	11.0 (2.7)	11.4 (2.5)	10.1 (2.6)	10.6 (3.0)	12.0 (2.9)	0.001^a^
Psychological	13.6 (2.4)	14.0 (2.3)	13.1 (2.4)	13.8 (2.5)	13.5 (2.6)	0.001^a^
Social	14.8 (3.4)	15.3 (3.2)	14.1 (3.4)	14.5 (3.8)	14.4 (3.8)	0.004^a^
Environment	14.2 (2.2)	14.5 (2.1)	13.9 (2.3)	14.0 (2.2)	14.3 (2.2)	0.025^a^

^a^The *p* value concerns the main effect of the ANOVA, and post hoc Tukey test showed a significant difference between the chronic pain diagnosis group and musculoskeletal diagnosis group in all domains and between the chronic pain diagnosis and the miscellaneous in the physical domain

The results of the regression analyses are summarized in Table 4.

**Table 4:** Results of the stepwise regression analyses with the different domains of the WHOQOL-BREF as dependent variables of rehabilitation outpatients (*n* = 542)

	B	SE B	Sig	95 % Confidence interval	
*Physical domain*					
Step 1					
Age	−0.025	0.008	0.003	−0.041	−0.008
Gender/male	0.367	0.249	0.141	−0.122	0.857
Education/low	−0.674	0.241	0.005	−1.147	−0.200
Living together	0.172	0.279	0.539	−0.376	0.719
Employed	0.408	0.236	0.084	−0.055	0.871
Step 2					
Chronic pain	−1.126	0.241	<0.001	−1.599	−0.653
*Psychological domain*					
Step 1					
Age	−0.015	0.008	0.056	−0.029	0.000
Gender/male	−0.140	0.225	0.535	−0.582	0.302
Education/low	−0.339	0.218	0.120	−0.766	0.089
Living together	0.760	0.252	0.003	0.265	1.255
Employed	0.636	0.213	0.003	0.218	1.054
Step 2					
Chronic pain	−0.788	0.219	<0.001	−1.219	−0.358
*Social domain*					
Step 1					
Age	−0.042	0.011	<0.001	−0.063	−0.021
Gender/male	−0.385	0.314	0.221	−1.002	0.232
Education/low	−0.350	0.304	0.250	−0.947	0.247
Living together	0.530	0.351	0.132	−0.161	1.220
Employed	0.997	0.297	0.001	0.413	1.580
Step 2					
Chronic pain	−0.916	0.307	0.003	−1.519	−0.312
*Environment domain*					
Step 1					
Age	−0.016	0.007	0.014	−0.029	−0.003
Gender/male	0.194	0.198	0.327	−0.195	0.583
Education/low	−0.945	0.191	<0.001	−1.321	−0.569
Living together	0.485	0.221	0.029	0.051	0.920
Employed	0.489	0.187	0.009	0.121	0.856
Step 2					
Chronic pain	−0.443	0.194	0.023	−0.825	−0.062

For gender the reference group was female, for education the reference group was middle or high education, for living together (consists of living with the family or a partner) the reference group was living alone, for employed the reference group was unemployment, and for chronic pain the reference group was the other diagnosis groups (musculoskeletal, neurological and miscellaneous)

*R* square of the regression models for physical domain was 0.082, for the psychological domain 0.070, for the social domain 0.073 and for the environment domain 0.091.

After correcting for patient characteristics, the diagnosis chronic pain contributed significantly to the regression equation in all domains.

**Fig. 1:**
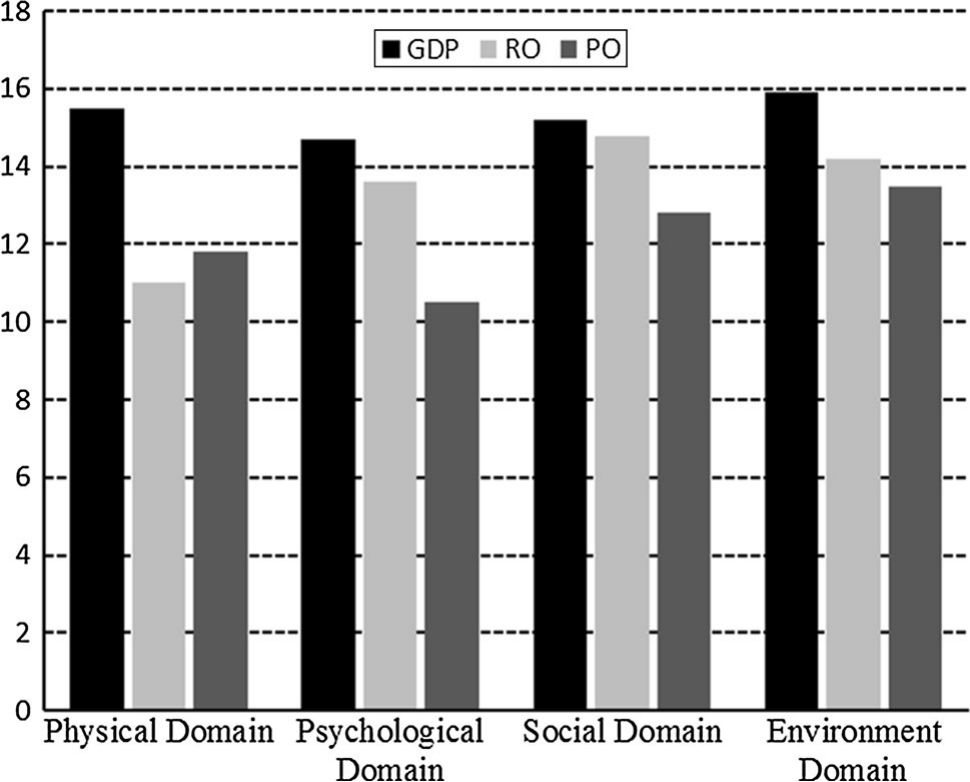
Comparison of domains of WHOQOL-BREF between the general Dutch population (GDP), rehabilitation outpatients (RO) included in the University Medical Center Groningen between 2008 and 2012 and the psychiatric outpatients (PO) (not adjusted for age and gender)

Compared to the general Dutch population, rehabilitation outpatient’s scores were significantly lower in all domains. Compared to psychiatric outpatients, rehabilitation outpatients’ scores were significantly higher except the physical domain (Fig. 1; Table 5). This comparison was not adjusted for age and gender.

**Table 5:** Comparison of domains of WHOQOL-BREF between the general Dutch population, rehabilitation outpatients seen in University Medical Center Groningen between 2008 and 2012 and the psychiatric outpatients (not adjusted for age and gender)

Domain	General Dutch population (*n* = 626^a^)		Difference	95 % CI^b^ lower	95 % CI upper	Rehabilitation outpatients (*n* = 542)		Difference	95 % CI lower	95 % CI upper	Psychiatric outpatients (*n* = 410)	
	Mean	SD				Mean	SD				Mean	SD
Physical	15.5	2.7	4.5	4.2	4.8	11.0	2.7	0.8	0.4	1.2	11.8	3.0
Psychological	14.7	2.2	1.1	0.4	1.2	13.6	2.4	−3.1	−3.4	−2.8	10.5	2.5
Social	15.2	2.9	0.4	0.0	0.8	14.8	3.4	−2.0	−2.4	−1.6	12.8	3.5
Environment	15.9	2.2	1.7	1.5	2.0	14.2	2.2	−0.7	−1.0	−0.4	13.5	2.5

^a^Owing to missing data, the number of participants from the general Dutch population differ per domain (range 619–626)

^b^Confidence interval

## Discussion

The current study aimed to provide normal values of the WHOQOL-BREF for outpatients in rehabilitation and to gain insight into the influence of diagnosis and patient characteristics on QOL. Compared to the general Dutch population, rehabilitation outpatients scored lower on all domains of WHOQOL-BREF, the physical domain most strongly. A higher age had a negative impact on QOL in all domains except the psychological domain. Unemployment had a negative impact on all domains except the physical domain. Living alone influenced the psychological and environmental domains negatively. Lower education influenced the physical and environmental domains negatively. Finally, gender had no significant influence on any domain.

### Diagnosis

In all four domains, the patients suffering from chronic pain were found to have a lower QOL than the musculoskeletal group. This influence was also significant after correcting for patient characteristics in all domains of WHOQOL-BREF. This finding corresponds with the concept that the emotional component plays an important role in chronic pain [24, 25].

### Rehabilitation patients, psychiatric patients and general Dutch population compared

Both psychiatric outpatients and rehabilitation outpatients scored lower on the physical domain than the general Dutch population, with the rehabilitation patients scoring the lowest. The psychiatric patients scored lower in the other three domains compared to the general Dutch population and to the rehabilitation outpatients. Further analyses revealed that the chronic pain patients had a lower score on the psychological domain but not as low as the psychiatric patients. The comparison with the psychiatric patients was not adjusted for age and gender. The comparison with the general Dutch population was not adjusted for age because data to do so were not available. Some age differences were present in our study. The mean age of the general Dutch population was 53.9 (SD 16.2), of the rehabilitation outpatients 41.0 (SD 14.0) and of the psychiatric outpatients 33.5 (SD 8.3). In a large WHOQOL-BREF study in the UK (*n* = 4628), including healthy people and people suffering from different health conditions, effects of age on WHOQOL-BREF scores were small [26]. There were no gender difference between the general Dutch population and the rehabilitation outpatients. These findings validate the assumption that rehabilitation patients primarily show difficulties coping with their physical problem and psychiatric patients with their mental problems.

### QOL as outcome measure/implications

The ability of the WHOQOL-BREF to evaluate change over time was investigated in a study within an outpatient rehabilitation setting. That study concluded that the questionnaire was a useful instrument for outcome measurement [17]. Also, statistically significant differences were found in all but the social domain, using raw data, between admission and discharge. Because raw data were used, it is difficult to assess the clinical impact of these differences. Moreover, the study used a small sample of 55 patients. WHOQOL-BREF has been used as a routine outcome measure, and changes were found in pre–post scores for some of 13 interventions investigated [26]. Only three of the interventions found a significant response in three or more domains: treatment as usual for depression, treatment as usual for arthritis and massage for chronic pain. Only four of the 13 treatments reported improvement in the psychological domain. The conclusion was that the responsiveness of the WHOQOL-BREF is limited or that the interventions were ineffective [26].

In the current study, QOL was measured once. The largest difference between the general Dutch population and the rehabilitation outpatients was in the physical domain, approximately 4 points on a 4–20 scale. The difference between the general Dutch population and rehabilitation outpatients was 1.1 point on the psychological domain and only 0.4 on the social domain. In our opinion, the differences in the psychological and social domain are small. This finding upholds one of the conclusions of the aforementioned study of a limited responsiveness [26].

### Strengths and limitations

The strength of the current study is the number of consecutive participants over a 5-year period. All referred patients were asked to participate. Of these participants, only 11 % were excluded. Limitation of the current study is a missing baseline measurement of QOL before the trauma or disease. However, these data cannot be obtained.

## Conclusion

In rehabilitation outpatients, scores on all WHOQOL-BREF domains were significantly lower than those of the general Dutch population. Therefore, the WHOQOL-BREF can be used to measure the subjective impact of their disease or injury in rehabilitation outpatients. A small but significant negative effect of increased age and unemployment was found on three domains, of living alone on two domains and of lower education also on two domains of QOL.

Patients with chronic pain were found to exhibit a significant lower QOL in all four domains when compared to the group of patients with musculoskeletal problems. The differences between the rehabilitation outpatients and the general Dutch population on the psychological and social domain are small.

## Abbreviations

WHOQOL-BREF: World Health Organization Quality of Life questionnaire abbreviated version
QOL: Quality of life
WHO: The World Health Organization
ICF: International classification of functioning, disability and health model
UMCG: University Medical Centre Groningen
ISCED: International Standard Classification of Education
